# Random field calibration with data on irregular grid for regional analyses: A case study on the bare carrying capacity of bats in Africa

**DOI:** 10.1002/ece3.10489

**Published:** 2023-09-10

**Authors:** Sena Mursel, Daniel Conus, Wei‐Min Huang, Javier Buceta, Paolo Bocchini

**Affiliations:** ^1^ Department of Civil and Environmental Engineering Lehigh University Bethlehem Pennsylvania USA; ^2^ Department of Mathematics Lehigh University Bethlehem Pennsylvania USA; ^3^ Institute for Integrative Systems Biology (I2SysBio), CSIC‐UV Paterna Spain

**Keywords:** Africa, autocorrelation, bare carrying capacity, bats, Ebola virus disease, irregularly spaced data, random fields

## Abstract

Many applications in science and engineering involve data defined at specific geospatial locations, which are often modeled as random fields. The modeling of a proper correlation function is essential for the probabilistic calibration of the random fields, but traditional methods were developed with the assumption to have observations with evenly spaced data. Available methods dealing with irregularly spaced data generally require either interpolation or computationally expensive solutions. Instead, we propose a simple approach based on least square regression to estimate the autocorrelation function. We first tested our methodology on an artificially produced dataset to assess the performance of our method. The accuracy of the method and its robustness to the level of noise in the data indicate that it is suitable for use in realistic problems. In addition, the methodology was used on a major application, the modeling of animal species connected with zoonotic diseases. Understanding the population dynamics of reservoirs of zoonotic diseases, such as bats, is a crucial first step to predict and prevent potential spillover of deadly viruses like Ebola. Due to the limited data on bats across Africa, their density and migrations can only be studied with probabilistic numerical models based on samples of the ecological bare carrying capacity ($K_{0}$). For this purpose, the bare carrying capacity was modeled as a random field and its statistics calibrated with the available data. The bare carrying capacity of bats was found to be denser in central Africa. This is because climatic and environmental conditions are more suitable for the survival of bats. The proposed methodology for random field calibration was shown to be a promising approach, which can cope with large gaps in data and with complex applications involving large geographical areas and high resolution.

## Graphical abstract

The manuscript presents a methodology to calibrate a random field using scarce and sparse data on an irregular grid. The methodology is used to assess random fluctuations in the bare carrying capacity of bats in Africa. These results are useful to model the zoonotic niche of many zoonotic diseases, such as Ebola.

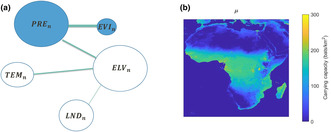

## 1 INTRODUCTION

In various fields such as finance, engineering, geology, epidemiology, and meteorology, the quantities involved in the problem are stochastic in nature and need to be modeled as random functions, that is, the collections of random variables defined over a parameter space (Pandey et al., 2022). In such cases, models require an appropriate description of the random variability in space and/or time of such quantities to capture their own behavior and the behavior of the systems that they might affect. The applications of random field representation vary from the hazard quantification to modeling of the disease risk and enable us to quantify the spatial and/or temporal variability of the quantities of interest (Christou & Bocchini, 2015; Kelsall & Eld, 2002). In the remainder of this article, the case of random fields (i.e., random functions defined over a spatial domain) will be considered, but the extension of the proposed methodology and findings to random processes (i.e., random functions defined over a temporal domain) is straightforward.

In most cases, the calibration of random fields leverages observations at a regularly spaced locations or at a grid of points. However, sometimes the values of these quantities are naturally observed at irregularly spaced locations due to several reasons, such as the nature of the random phenomenon that leads it to occur at random locations (e.g., locations of the certain level of corrosion in a steel beam), or the fact that the locations of the measurements cannot be chosen completely arbitrarily (e.g., locations of weather stations). The application that motivated this study and the development of the proposed methodology consists of irregularly spaced observations of the population of animals to estimate their population density. Studies on these topics typically combine datasets from multiple sources in the literature, which obviously were not systematically coordinated. But even within a single campaign, when there is a deliberate effort to collect presence data on wildlife, these observations occur at random locations.

In ecology, conservation biology, epidemiology, and biodiversity, modeling species geographic distributions has a substantial role on applications such as the management of threatened species, prediction of impacts of climate change, biological invasions, and further disease control (Guillera‐Arroita et al., 2015). In particular, zoonoses (i.e., zoonotic diseases) are infectious diseases that are transmitted from animals to humans and are responsible for most outbreaks worldwide (Jones et al., 2008). Since about 75% of emerging infectious diseases are zoonoses (mostly originated in wildlife) and have become a growing threat for the public health, global economy, and security (Smith & Wang, 2013), understanding the population dynamics of a species which is the carrier and reservoir of a disease is of major importance. In general, mechanisms of disease emergence are complex and identifying agencies behind the transmission of zoonoses and dynamics of interactions between wildlife and human population need to be a persistent study area for researchers and public health authorities to combat potential and existing outbreaks. In particular, one of the most lethal zoonoses, Ebola virus disease (EVD), commonly referred to as “Ebola,” is a zoonotic disease caused by a filovirus, *Ebolavirus* (Martines et al., 2015). Evidence has suggested that fruit bats may act as a main reservoir of Ebolavirus (Leroy et al., 2005; Olival & Hayman, 2014). As the Centers for Disease Control (CDC) pointed out, an understanding of hemorrhagic fever incidence will not be possible until the ecology of the reservoir is established and its zoonotic niche correctly modeled. The interest is not limited to Ebola. For instance, Nipah is another highly infectious zoonotic virus that can cause severe illness in humans and fruit bats were also identified as the natural reservoir hosts (Chua, 2003). Thus, understanding the key factors of bat migration and nesting can enable us to know the bat population density and this will serve as a first important condition for the prevention of these zoonotic diseases.

At the moment, data on bat presence and density is very limited and difficult to acquire, so the very first step to study the zoonotic niche is to simulate the geographic distribution and density of the bat population, preferably at high resolution. Here, to address this problem, our aim is to estimate the environmental bare carrying capacity for bats ($K_{0}$), defined as the average population size of a species that can be sustained by the surrounding environment, as a function of environmental parameters (e.g., temperature, vegetation index) in the entire continent of Africa. However, the observation data on species' of bats are very scarce and scattered. Modeling the bare carrying capacity for bats across the African continent at a moderate to high resolution requires to consider the uncertain nature of the phenomenon. Therefore, this problem belongs to the class discussed earlier, where a quantity can be modeled as a random field and its calibration can be done only using observations at irregular locations. The modeling of a correlation function is one of the key steps in the calibration of a random field (Risser & Calder, 2015). The structure of the observations data does not allow us to employ conventional methods to estimate the autocorrelation because the data are not on a regular grid.

Hence, this study aims to describe an innovative grid‐less approach to estimate the autocorrelation function. This novel approach is both simple and effective and we used the observations on bats across Africa as the focus of this study to illustrate the framework. With the methodology described in this article, it is possible to calibrate a random field model and then generate random samples of the bare carrying capacity, which can be used in a simulation‐based approach to study the probabilistic distribution of infected bats. An important feature of the developed model is that it can be modulated by the seasonal and long‐term variability of the climatic and environmental parameters. For instance, in addition to the annual mean bare carrying, specific values of the mean of $K_{0}$ have been calibrated for different months of the year. Specific samples of $K_{0}$ for each month can be obtained by superimposing the random noise onto the appropriate mean surface. This aspect of the proposed model is crucial because it enables the generation of bare carrying capacity samples based on present and future climatic conditions, obtained by downscaling of global circulation models that account for climate change.

After this introduction, Section 2.1 of the article defines the random field model that we used and Sections 2.2 and 2.3 introduce the proposed methodology for calibration of the autocorrelation function with irregularly spaced data. Section 2.4 presents the application of the methodology to a benchmark example, to assess the accuracy of the proposed methodology and its robustness to noise. Section 2.5 showcases the main application to the bare carrying capacity, to show how the methodology can scale to address large‐scale and high‐resolution datasets. Section 3 presents and discusses the results obtained for both applications. Finally, Section 4 summarizes the conclusions and main findings of the study.

## 2 METHODS

### 2.1 Random fields background

A random field, $f_{0}\left(x,\theta \right)$, is defined as a collection of random variables indexed by spatial parameter x∈Ω, where Ω is the predefined geometry of spatial domain and θ∈Θ is an outcome from the sample space Θ. A random field is defined by its mean function $\mu \left(x\right)=E\left[f_{0}\left(x\right)\right]$, variance $\sigma^{2}\left(x\right)=E\left[{\left(f_{0}\left(x\right)-\mu \left(x\right)\right)}^{2}\right]$, and the autocorrelation function (ACF), $R_{f_{0}f_{0}}\left(x,x^{\prime }\right)=E\left[\left(f_{0}\left(x\right)-\mu \left(x\right)\right)\left(f_{0}\left(x^{\prime }\right)-\mu \left(x^{\prime }\right)\right)\right]/\left(\sigma \left(x\right)\sigma \left(x^{\prime }\right)\right)$. If the field is homogeneous (i.e., stationary in space), the mean and the variance of the field are independent of the spatial parameter, thus they are constants, and the autocorrelation function is only dependent on the distance $\left(x-x^{\prime }\right)$, that is, lag (Crandall & Mark, 1963). While many theoretical models of the ACF are available in the literature, in practical problems, it is very often found that a negative exponential decay captures well the ACF:

(1) $$R_{f_{0}f_{0}}\left(x,x^{\prime }\right)=\prod_{i=1}^{\dim}\exp\left[{\left(-\frac{\left\|x_{i}-{x_{i}}^{’}\right\|}{b_{i}}\right)}^{{\mathrm{pow}}_{i}}\right]$$

where dim is the dimensionality of the spatial domain (e.g., 2 for a geographic region), ${\mathrm{pow}}_{i}$ are parameters to be chosen depending on the problem at hand (most often equal to 1 or 2), ‖⋅‖ is a norm (usually the Euclidean norm) and $b_{i}$, $\left(i=1,\ldots ,\dim\right)$ are the correlation lengths for each dimension. Correlation length is an indicator showing how much nearby points in the field are correlated. The correlation length is an important parameter with the physical meaning of indicating how far the value of the field at one location can be correlated with values of the field at other locations.

Ideally, the complete probabilistic description of a random field requires to consider infinite number of samples, which is clearly impractical. However, it can be proved that most homogeneous fields are also ergodic, which means that each individual sample reflects all the statistical properties of the field. Unfortunately, tests of ergodicity are weak and data intensive. Therefore, it is common in practical applications to test the field for homogeneity and then assume ergodicity if the homogeneity test is passed. In this case, a single sample of the field is sufficient to estimate the statistical characteristics of the whole field. It will be shown in Section 3 that the fields considered in this study are homogeneous and only one sample of observations is available for the main application, so the assumption of ergodicity is necessary and justified.

### 2.2 Irregularly spaced data

As mentioned, it is often found that the values of the geospatial sample of a random field are available at an irregular grid of locations. When such data represent measurements of a random field, the calibration of its properties becomes challenging, because traditional techniques assume to have values of the sample at regular frequencies in time and/or space. To circumvent the problem of irregularly spaced data, several remedies have been proposed in the literature. One of the most intuitive solutions is the interpolation of the original data to obtain an evenly spaced series (Scargle, 1989). Yet, the success of the interpolation in representing the original data depends on the form of the interpolating function, the smoothness of the original data series and the presence of large gaps in the data. Also, this method creates bias because the interpolated data points are no longer independent. In addition, converting unevenly spaced data to an equally spaced data reduces and dilutes the information content of a dataset. If two consecutive observations are close to each other, data points need to be omitted, which causes statistical inference to be less efficient. Redundant data points are introduced if consecutive observations lie far apart, thus causing another dimension of bias. In the case of the bare carrying capacity, the presence of very large gaps prevents the use of interpolation, because it could lead to disastrous results. For irregularly spaced data that are relatively homogeneously distributed, another possible solution is the downsampling of the space or time lags used to define the autocorrelation function (i.e., the auto‐covariance function, when the process/field has mean 0) (Edelson & Krolik, 1989).

In this case, unequally spaced lags $\left(\xi_{1},\xi_{2}\right)$ are grouped into equally spaced bins or intervals. For calculation of the auto‐correlation function (ACF), all values of the product $f_{0}\left(x_{1}^{j},x_{2}^{j}\right)\cdot f_{0}\left(x_{1}^{k},x_{2}^{k}\right)$ associated with lags within each lag interval are averaged, where $f_{0}\left(x_{1}^{j},x_{2}^{j}\right)$ is the value of the sample, at location j, $f_{0}\left(x_{1}^{k},x_{2}^{k}\right)$ is the value at k, and the j−k lags are $\left(\xi_{1}=|x_{1}^{j}-x_{1}^{k}|,\xi_{2}=|x_{2}^{j}-x_{2}^{k}|\right)$. This method gives a naturally smoothened estimate of the autocorrelation function. If the data itself have large gaps, large lag intervals are required to estimate the autocorrelation function and the resulting level of smoothing becomes unacceptable (Craymer, 1998). More sophisticated solutions for unequally spaced data based on interpolation and approximation like the two mentioned above were developed, but they all share the issue that they cannot deal with large gaps in the original dataset.

Other methods were proposed, based on the least square transform and its inverse (Craymer, 1998; Vaníček, 1971). These methods can estimate spectrum and autocorrelation accurately, but they are not computationally efficient and currently they have only been formulated for and applied to one‐dimensional problems, so they would need to be extended to the multidimensional case for our study. Because such methods were already shown to have efficiency issues even for one‐dimensional cases, we expect that the issues will be exacerbated for multidimensional problems and we did not investigate these methods further.

### 2.3 Proposed methodology for the calibration of random fields with irregular grid

The proposed methodology is presented for the case of two‐dimensional (2D) random fields with observations available over an irregular grid, but the same procedure can be directly applied to other fields with any number of dimensions. A random field $K_{0}\left(x,\theta \right)$ can be seen as the combination of a deterministic mean surface $\mu \left(x\right)$ and the fluctuations around the mean surface due to various reasons, including but not limited to the modeling error, limited information about the field, and imprecise estimation of input variables. If the mean surface is subtracted from the field, the resulting “residuals” can be modeled as another two‐dimensional random field $f_{0}\left(x,\theta \right)$ which has mean equal to zero by construction, and in general is non‐Gaussian. This can be represented as Equation (2).

(2) $$K_{0}\left(x,\theta \right)=\mu \left(x\right)+f_{0}\left(x,\theta \right)$$

In the remainder of this section, to simplify the notation, the dependence on the random outcome θ will be omitted and the dependence on the location vector x will me made explicit for the two‐dimensional case: $x_{1},x_{2}$.

If only one sample f of field $f_{0}$ is available, a preliminary test of homogeneity needs to be conducted. If the test is positive, then it is necessary (and usually reasonable in practice) to assume ergodicity of the field to proceed with the calibration. If the field is not homogeneous, then it is certainly not ergodic and the calibration is impossible with a single sample. In such case, more samples need to be collected and/or the domain needs to be partitioned into region where the field is homogeneous. The next step is to determine the marginal distribution of the field. This can be done with parametric or non‐parametric methods (Clifford, 1990). If the distribution is non‐Gaussian, then the sample is mapped into an associated sample g with Gaussian distribution. For the estimation of the ACF for irregularly spaced data, all the pairs $\left(j,k\right)$ of locations at which data are available are listed. For every pair, the distances in both directions (i.e., the lags) and the product of the values of the sample are calculated. These three quantities (i.e., distance along dimension 1, distance along dimension 2, and the product of the values) serve as an empirical estimate of the autocorrelation function (EACF). If the data were on a regular grid, all the products clustered at each specific value of the lags would be averaged. However, since the spacing is irregular in this study, the vast majority of the products correspond to different values of the lags. Hence, instead of averaging, a least square regression is proposed, to identify and fit an appropriate closed form expression for the autocorrelation function, choosing among a set of potential models like Equation (1). This approach provides a natural way to cope with large gaps in the EACF estimate and an irregular distribution of the values, while preserving a familiar framework for the ACF estimation. The following steps summarize the described procedure.

#### 2.3.1 Input

The input consists of a set of observations $K_{\mathrm{obs}}$ at random locations $\left({\tilde{x}}_{1},{\tilde{x}}_{2}\right)$. Moreover, it is assumed that a set of additional variables $X_{j}$ are known over the domain of study, and somehow related with the investigated quantity $K_{0}$.

#### 2.3.2 Step 0

A necessary preliminary step consists in estimating the mean surface $\mu \left(x_{1},x_{2}\right)$. Any data‐driven approach for predicting $\mu \left(x_{1},x_{2}\right)$ can be chosen. However, the remainder of this study focuses only on regression models. A regression analysis can be made in such a way to connect the observations $K_{\mathrm{obs}}$ with the known values of the features $X_{j}$. Multiple types of regression models in the $\left(x_{1},x_{2}\right)$ coordinates should be considered, such as multiple linear regression (MLR), multiple linear regression with cross terms (GLC) and second‐order polynomial regression with cross terms (GPC). For the selection of the best model, the adjusted *R* ^2^ was used as the metric of accuracy. The *regsubsets* function of the contributed *R* package *leaps* can be used to serve this purpose (R Core Team, 2013). In particular, *regsubsets* performs an exhaustive search for the best subset of terms in the model to make the prediction, for any given number of terms. Then, to select the number of terms yielding the best compromise between accuracy and simplicity, twofold cross validation should be performed. For each number of terms in the model, the data should be randomly split into two sets for training and testing. In our experience, the best results were obtained with a 50%–50% split. The process should be repeated for a large number of random splits of the data, and the mean adjusted *R* ^2^ of the testing sets should be used to determine when the accuracy of the model reaches a plateau, meaning that the use of additional terms does not contribute to the accuracy in a meaningful way. The results of this regression constitute the estimate of the mean surface $\hat{\mu }\left(x_{1},x_{2}\right)$. The observations of the random fluctuations are then computed as:

(3) $$f_{\mathrm{obs}}\left({\tilde{x}}_{1},{\tilde{x}}_{2}\right)=K_{\mathrm{obs}}\left({\tilde{x}}_{1},{\tilde{x}}_{2}\right)-\hat{\mu }\left({\tilde{x}}_{1},{\tilde{x}}_{2}\right)$$

The single sample $f_{\mathrm{obs}}$ needs to be tested for homogeneity (as a proxy for ergodicity). This can be done by splitting the spatial domain into smaller regions and assessing the probabilistic characteristics of each region. These regions should collectively cover the entire domain, and it is good practice to include also some regions overlapping with each other to perform a better evaluation. The empirical Cumulative Distribution Function (CDF) of $f_{\mathrm{obs}}$ should be computed over the entire domain (${\hat{F}}_{\mathrm{NG}}$) and in each region. The maximum discrepancy between the global CDF and the CDF in each region as computed by the Kolmogorov–Smirnov test should be smaller than a predefined threshold. In theory, a test to assess the homogeneity of the second order statistics (e.g., ACF) should be performed too, but in practice when the observations are scarce to assess the ACF over the entire domain, it will be impossible to estimate the ACF in the smaller regions. If the test of homogeneity is passed, field $f_{0}$ is assumed to be homogeneous and ergodic.

#### 2.3.3 Step 1

If the global CDF of $f_{\mathrm{obs}}$ is non‐Gaussian, the values of all observations at their locations $\left({\tilde{x}}_{1},{\tilde{x}}_{2}\right)$ are mapped to the corresponding standard Gaussian distribution, using the inverse Nataf transformation (Oger & Saporta, 1991; Shields et al., 2011):

(4) $$g_{\mathrm{obs}}\left({\tilde{x}}_{1},{\tilde{x}}_{2}\right)=F_{G}^{-1}\left\{{\hat{F}}_{\mathrm{NG}}\left[f_{\mathrm{obs}}\left({\tilde{x}}_{1},{\tilde{x}}_{2}\right)\right]\right\}$$

where ${\hat{F}}_{\mathrm{NG}}$ is the empirical cumulative distribution function of the sample, and $F_{G}^{-1}$ is the inverse standard Gaussian cumulative distribution function. If $f_{0}$ is itself Gaussian, then we can just set $g_{\mathrm{obs}}=f_{\mathrm{obs}}$.

#### 2.3.4 Step 2

Observations of the EACF $R_{\mathrm{obs}}\left(\xi_{1},\xi_{2}\right)$ of the zero‐mean random field $g_{0}$ are calculated as the products of the values of $g_{0}$ observed at spaces separated by the distances $\left(\xi_{1},\xi_{2}\right)$ for each pair:

(5) $$R_{\mathrm{obs}}\left(\xi_{1},\xi_{2}\right)=g_{\mathrm{obs}}\left({\tilde{x}}_{1}^{j},{\tilde{x}}_{2}^{j}\right)\cdot g_{\mathrm{obs}}\left({\tilde{x}}_{1}^{k},{\tilde{x}}_{2}^{k}\right)\;\forall \;j,k$$

where $\xi_{1}=|x_{1}^{j}-x_{1}^{k}|$ and $\xi_{2}=|x_{2}^{j}-x_{2}^{k}|$. If multiple pairs of locations happen to be separated by the same distances $\left(\xi_{1},\xi_{2}\right)$, they will generate multiple observations of the surface $R_{\mathrm{obs}}$ at the same coordinates. It should be noted that the field has been assumed to be homogeneous, so even if the locations $\left({\tilde{x}}_{1}^{j},{\tilde{x}}_{2}^{j}\right)$ and $\left({\tilde{x}}_{1}^{k},{\tilde{x}}_{2}^{k}\right)$ appear in the right‐hand side of Equation (5), the left‐hand side depends only on the distances $\left(\xi_{1},\xi_{2}\right)$.

#### 2.3.5 Step 3

Several analytical expressions which are commonly used to describe the ACF are fitted to $R_{\mathrm{obs}}\left(\xi_{1},\xi_{2}\right)$ and the best fit is selected by visual inspection and by checking the adjusted $R^{2}$ as a measure of accuracy. If multiple observations of $R_{\mathrm{obs}}$ are available at some locations, the regression process will automatically consider all of them. The fitted surface with its analytical expression ${\hat{R}}_{g_{0}g_{0}}\left(\xi_{1},\xi_{2}\right)$ is the estimate of the ACF of the random field.

#### 2.3.6 Step 4

By applying the Wiener–Khinchin theorem, the corresponding spectral density function (SDF) is calculated because it is necessary for the generation of samples.

(6) $${\hat{S}}_{g_{0}g_{0}}\left(\kappa_{1},\kappa_{2}\right)=\frac{1}{{\left(2\pi \right)}^{2}}\iint_{-\infty }^{\infty }{\hat{R}}_{g_{0}g_{0}}\left(\xi_{1},\xi_{2}\right)\cdot \exp\left(-i\kappa_{1}\xi_{1}-i\kappa_{2}\xi_{2}\right)d\xi_{1}d\xi_{2}$$

where ${\hat{S}}_{g_{0}g_{0}}$ is the estimate SDF of the field $g_{0}$; $\kappa_{1}$ and $\kappa_{2}$ are the wave numbers in the two spatial dimensions.

Once the calibration process described above in Steps 0–4 is completed, its results can be used to generate samples of the investigated field. In the past few decades, a variety of algorithms to simulate non‐Gaussian stochastic processes and fields were developed. The spectral representation method (SRM) has emerged as one of the most popular and widely used techniques among those (Shields & Deodatis, 2013). It was firstly developed for Gaussian random processes and then extended to non‐Gaussian stochastic processes by combining translation process theory (Grigoriu, 1984). In our work, we chose to use the SRM because of its ease of implementation, accurate results, and popularity (Liu et al., 2016). In particular, for the case of a two‐dimensional and univariate quadrant field (i.e., special case of homogeneous random field), the simulation formula was presented by Christou and Bocchini (2014):

(7) girΔx1,sΔx2=2expi2πn1rM1+i2πn2sM2⋅Re∑n1M1−1∑n2M2−12Sg0g0κ1n1,κ2n2Δκ1Δκ2eiϕn1n21i+2⋅Re∑n1M1−1∑n2M2−12Sg0g0κ1n1,κ2n2Δκ1−Δκ2eiϕn1n22i⋅expi2πn1rM1−i2πn2sM2

where:

(8) $$\kappa_{1_{n_{1}}}=n_{1}\Delta \kappa_{1};\;\kappa_{2_{n_{2}}}=n_{2}\Delta \kappa_{2}$$

(9) $$\Delta \kappa_{1}=\frac{\kappa_{1u}}{N_{1}};\;\Delta \kappa_{2}=\frac{\kappa_{2u}}{N_{2}}$$

(10) $$M_{1}\geq 2N_{1};\;M_{2}\geq 2N_{2}$$

(11) $$r=0,1,\ldots ,M_{1}-1;\;s=0,1,\ldots ,M_{1}-1$$

and $\phi_{n_{1}n_{2}}^{\left(1\right)\left(i\right)},\phi_{n_{1}n_{2}}^{\left(2\right)\left(i\right)}$ are the i‐th sample set of independent random phase angles uniformly distributed over the interval $\left[0,2\pi \right]$. In Equation (9), $\kappa_{1u}$ and $\kappa_{2u}$ are the upper cutoff wave numbers corresponding to the $x_{1}$ and $x_{2}$ axes in the space domain, respectively. The spectrum for wave numbers exceeding $\kappa_{1u}$ and $\kappa_{2u}$ is considered to be equal to 0 for practical purposes. The criteria in Equation (10) should be satisfied in order to prevent possible aliasing effects in sampling. Due to its significant computational benefits, the SRM algorithm based on Fast Fourier Transform (FFT) was used in this study (Shinozuka & Deodatis, 1996). Using Equation (6) with the estimate spectrum ${\hat{S}}_{g_{0}g_{0}}$ results in the generation of Gaussian samples of $g_{0}$. Such samples are then mapped to samples of $f_{0}$ using the direct Nataf transformation and the estimated distribution ${\hat{F}}_{\mathrm{NG}}$:

(12) $$f^{\left(i\right)}\left(x_{1},x_{2}\right)={\hat{F}}_{\mathrm{NG}}^{-1}\left\{F_{G}\left[g^{\left(i\right)}\left(x_{1},x_{2}\right)\right]\right\}$$

Finally, samples of $f_{0}$ can be superimposed to the estimated mean surface $\hat{\mu }$ to obtain the desired samples of $K_{0}$:

(13) $$K^{\left(i\right)}\left(x_{1},x_{2}\right)=\hat{\mu }\left(x_{1},x_{2}\right)+f^{\left(i\right)}\left(x_{1},x_{2}\right)$$

### 2.4 Application on a benchmark example

To assess the ability of the proposed methodology to perform its function, even in the case of noisy and sparse input data, a benchmark application was devised and used for testing. Because we had deliberately defined all the parameters and the probabilistic characteristics of the benchmark application, it was possible to assess the accuracy of the proposed methodology.

The benchmark example consists of a non‐homogeneous, non‐Gaussian, two‐dimensional random field $K_{0}\left(x_{1},x_{2}\right)$. First, a surface $\mu \left(x_{1},x_{2}\right)$ was defined over the 2D spatial domain, to serve as the non‐homogeneous mean of $K_{0}$. It was assumed that four features $X_{1}\left(x_{1},x_{2}\right),\;X_{2}\left(x_{1},x_{2}\right),\;X_{3}\left(x_{1},x_{2}\right)$, and $X_{4}\left(x_{1},x_{2}\right)$ are fully known over the spatial domain, and the investigated field $K_{0}$ is known to be somehow related to these four features. Therefore, the mean surface μ was defined as an arbitrary nonlinear combination of $X_{1},\;X_{2},\;X_{3}$, and $X_{4}$. Then, a single sample $f\left(x_{1},x_{2}\right)$ of a lognormal, zero mean, homogeneous, two‐dimensional random field $f_{0}\left(x_{1},x_{2}\right)$ was generated to represent the random fluctuations of $K_{0}$ around the mean μ. The sum of the mean surface and the fluctuations around the mean yielded a single sample K of the benchmark random field $K_{0}$:

(14) $$K\left(x_{1},x_{2}\right)=\mu \left(x_{1},x_{2}\right)+f\left(x_{1},x_{2}\right)$$

The values of the sample K were generated at a regular and dense grid of points in the 2D spatial domain. To simulate observations at a sparse and irregular grid, a small subset of grid points with coordinates $\left({\tilde{x}}_{1},{\tilde{x}}_{2}\right)$ were selected randomly and only the values of the benchmark samples at those locations were used as input of the proposed methodology for calibration. To simulate imperfect observations and measurement errors, a random noise was superimposed to the values of the simulated sample before using them as input for the calibration. The noise consisted in a random perturbation $z\left({\tilde{x}}_{1},{\tilde{x}}_{2}\right)$ with Gaussian distribution, zero mean, variance equal to a predefined percentage of the variance of $f\left(x_{1},x_{2}\right)$, and sampled independently for each location of the field.

(15) $$K_{\mathrm{obs}}\left({\tilde{x}}_{1},{\tilde{x}}_{2}\right)=\mu \left({\tilde{x}}_{1},{\tilde{x}}_{2}\right)+f\left({\tilde{x}}_{1},{\tilde{x}}_{2}\right)+z\left({\tilde{x}}_{1},{\tilde{x}}_{2}\right)$$

These values of $K_{\mathrm{obs}}$ simulate sparse and noisy observations of the field, and they were then fed as input to the methodology described in Section 2.3. As discussed in Step 0, the mean surface was assessed first, by doing a regression on the available noisy observations $K_{\mathrm{obs}}$. MLR, GLC, and GPC were used as candidate regression models. Then, Steps 1–4 described in Section 2.3 were followed to determine the ACF and SDF of the fluctuations.

The results of the calibration were then assessed. In addition to the evaluation of the adjusted *R* ^2^, the functional form and parameters of $\hat{\mu }$ were compared to the (known in this benchmark) functional form and parameters of μ to estimate the accuracy of the regression. For the marginal distribution of the fluctuations field, the Kolmogorov–Smirnov (KS) index was used as a metric of uniform convergence of the empirical CDF of $f_{\mathrm{obs}}$ to the CDF of $f_{0}$ (known in this benchmark). For the ACF, the value of adjusted *R* ^2^ of the fitted ACF ${\hat{R}}_{g_{0}g_{0}}$ were calculated and the correlation lengths in latitude and longitude were compared to the true values (known in this benchmark).

The whole procedure was repeated for various severity levels of the noise (i.e., variance of z equal to 0%, 1%, 3%, 5%, 10%, 30%, and 100% of the variance of $f_{0}$), to assess the robustness of the estimation protocol to noisy data.

### 2.5 Application to the bare carrying capacity of bats in Africa

At the moment, data on bat presence and density is very limited and difficult to acquire, so the very first step to study the zoonotic niche is to simulate the bat population, while accounting for the uncertainty present in these projections. Such simulation is mostly driven by the carrying capacity $K_{0}$, which needs to be modeled on the basis of sparse and scarce observations of bat presence. As for any field, the value of $K_{0}$ for bats as a function of longitude, latitude, and time can be represented using two components: a mean value and a random fluctuation around the mean. In this case, the mean value can be modeled as a function of known enviroclimatic factors because they influence available resources such as food and nesting areas, whereas the fluctuations can be modeled as a random field. Recently, a deterministic version of the model was published by Fiorillo et al. (2018). In this article, an improved, probabilistic version of the model is presented, to capture the model error and the intrinsic uncertainties in the environmental characteristics. Unfortunately, the information on bat presence and absence is not available on a regular grid, but at a set of locations spread over Africa without any particular pattern. With the methodology described in Section 2.3, the new model can be calibrated.

In this study, we account for four different species of bats in the sub‐Saharian region of Africa: *Eidolon helvum*, *Macronycteris gigas* (*formerly known as Hipposideros gigas*), *Hypsignathus monstrosus*, and *Rousettus aegyptiacus* (IUCN, 2015). The selection of these specific species was based on their possible involvement in filovirus infections, their habit to cluster in large colonies, and the availability of quantitative data on their presence (Fiorillo et al., 2018; Ohimain, 2016). Despite the existence of other bat species in the region, Fiorillo et al. (2018) showed that discarding other species is an unbiased and realistic approach. The maximum colony sizes of the mentioned species were considered to be 500,000 for *Eidolon helvum*, 1000 for *Macronycteris gigas*, 100 for *Hypsignathus monstrosus*, and 5000 for *Rousettus aegyptiacus* bats (Fiorillo et al., 2018). Evidently, *Eidolon helvum* dominates quantitatively the distribution of observed bats, but other species were also included in the study to be more realistic. Colonies of *Eidolon helvum* were shown to have a foraging radius between 10 and 20 km (Richter & Cumming, 2006, 2008), and we took the radius of 30 km, twice of the average foraging distance, as the upper‐bound for the total area occupied by a colony. The spatial densities, thus, were calculated by dividing the size of the colony by the area of influence for each species. Similar calculations were done for all considered species and the results are in Table 1. The resulting spatial densities were assumed to be observations of the bare carrying capacity at locations where the presence of a certain species was detected (Bergmans, 1989). Additionally, absence of bat species for places where it is certainly known that no bats can survive (i.e., deserts, large bodies of water) was also added to the dataset, to have a more realistic picture (Fiorillo et al., 2018; IUCN, 2015). Similarly, artificial observations were added in general areas of known presence (Fiorillo et al., 2018; IUCN, 2015). The result is a set of observations $K_{\mathrm{obs}}$ at sparse locations $\left({\tilde{x}}_{1},{\tilde{x}}_{2}\right)$ (see folder S3 in Mursel et al., 2023).

**TABLE 1:** Species and their population densities.

Name of species	Bare carrying capacity for species
*Eidolon helvum*	176.84
*Macronycteris gigas*	0.35
*Hypsignathus monstrosus*	0.035
*Rousettus aegyptiacus*	1.77

Environmental and geographical data (i.e., features) were gathered using the Google Earth Engine tool to access the databases from the NASA Land Processes Distributed Active Archive Center (LP DAAC) at the USGS Earth Resources Observation and the Science (EROS) Center, Sioux Falls, South Dakota (NASALP‐DAAC, 2022). For this study, we considered eight variables: enhanced vegetation index (EVI, dimensionless), precipitation (PRE, in mm/5 days), daily air temperature (TMP, in °C), land cover index (LND, dimensionless), ground elevation (ELV, in m), human population density (POP, in individuals/km^2^), latitude (LAT, in °), and longitude (LON, in °) (see folder S1 in Mursel et al., 2023). It should be noted that all the variables but LND are quantitative. However, LND is a categorical variable with subcategories sorted based on the density of vegetation and they can be assigned a corresponding quantitative value from 0 to 15 (i.e., 0 is water and 15 is an area with high density of vegetation) to be consistent with other variables.

The observations of these environmental features were averaged over 10 years (from 2000 to 2010) at a resolution of 10 km × 10 km. These variables were then divided by their maximum value in the set, to make them dimensionless and normalized in the interval [0, 1], and to create the input set used for analysis (i.e., ${\mathrm{EVI}}_{n}$, ${\mathrm{PRE}}_{n}$, ${\mathrm{TMP}}_{n}$, ${\mathrm{LND}}_{n}$, ${\mathrm{ELV}}_{n}$, *POP*_*n*_, *LAT*_*n*_, and ${\mathrm{LON}}_{n}$). The maximum values of each variable were the following: $EVI_{\max}$ = 1, $PRE_{\max}$ = 100 mm/5 days, $TEM_{\max}$ = 50°C, $LND_{\max}$ = 15, $ELV_{\max}$ = 5000 m, $POP_{\max}$ = 400 individuals/km^2^, $LAT_{\max}$ = 40°, and $LON_{\max}$ = 50°.

As the relationship between $K_{0}$ and environmental variables cannot be defined mechanistically, a regression model was used instead. As opposed to other data‐driven models, a simple regression offers a direct interpretation of the relationship between output (i.e., $K_{0}$) and inputs (i.e., environmental variables). In particular, we focused on three types of regression models already discussed in Section 2.3: MLR, GLC, and GPC. For this application, the cross‐validation with process was repeated for 500 random splits of the data between testing and training. Once the estimate mean surface was available, the statistics of the residuals random field $f_{0}$ were computed using the procedure explained in Section 2.3.

The data for environmental variables are at a resolution of 10 km × 10 km, thus the samples of random field $g_{0}$ (and in turn $f_{0}$ and $k_{0}$) were generated at the same resolution, to be consistent. Any number of different samples can be generated using the presented methodology.

## 3 RESULTS

### 3.1 Benchmark example

For this study, the four features $X_{1},X_{2},X_{3}$, and $X_{4}$ were arbitrarily defined as follows:

(16) $$\left\{\begin{array}{l} X_{1}\left(x_{1},x_{2}\right)=\pi^{2}\cdot \cos\left(x_{1}/2+x_{2}/4\right)\\ X_{2}\left(x_{1},x_{2}\right)=\sqrt{\left(\exp\left(x_{1}/10+x_{2}/4\right)\right)}/400\\ X_{3}\left(x_{1},x_{2}\right)=\left(x_{1}^{2}/20+\sqrt{x_{2}}/100\right)\cdot \cos\left(2\pi \cdot x_{1}/100\right)/10\\ X_{4}\left(x_{1},x_{2}\right)=x_{1}\cdot x_{2}/300 \end{array}\right.$$

with $x_{1},x_{2}\in \left[0,40\right]$.

The spatial coordinates were discretized at a resolution of 0.2 × 0.2 in the range [0, 40] × [0, 40]. The mean surface $\mu \left(x_{1},x_{2}\right)$ was obtained as a nonlinear combination of the four features, as shown in Equation (17).

(17) $$\mu \left(x_{1},x_{2}\right)=10X_{1}+6X_{1}\cdot X_{3}+8X_{4}-4X_{2}$$

**FIGURE 1:**
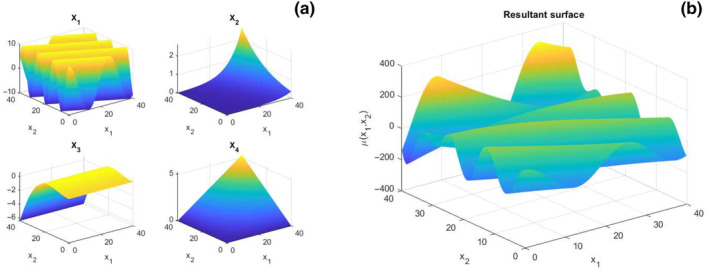
Four baseline features (a), and resultant mean surface using Equation (17) (b) for the benchmark example.

Figure 1 illustrates the four features and the resulting mean surface.

The fluctuations around the mean $f_{0}$ have a shifted lognormal distribution with minimum value equal to −35, mean 0, and standard deviation 16. The ACF of the fluctuations has the expression in Equation (1), where parameter pow is 1, and the correlation lengths $b_{1}$ and $b_{2}$ are 10 and 12, respectively.

The level of noise z was set to zero in this preliminary phase of the analysis.

**FIGURE 2:**
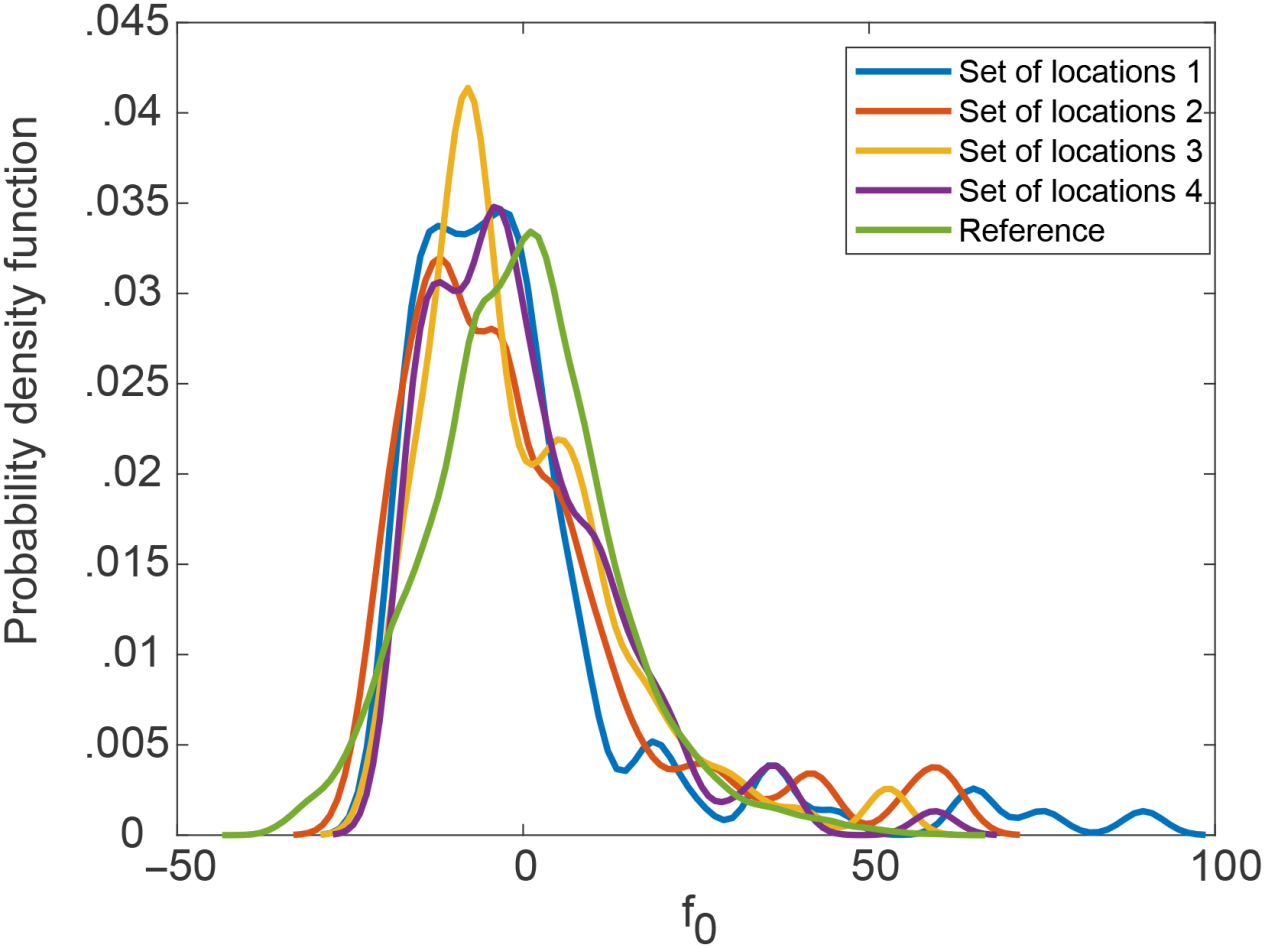
Probability density functions of residuals for different set of locations.

We selected 100 random locations $\left({\tilde{x}}_{1},{\tilde{x}}_{2}\right)$ to have observations at a sparse and irregular grid. The choice of the number of locations is representative of practical situations with a very small number of observations. It should be noted that 100 is even lower than the number of observations available for bats in the main application of this study (Section 3.2). The entire procedure discussed in this section was repeated four times with different sets of random locations $\left({\tilde{x}}_{1},{\tilde{x}}_{2}\right)$ for the observations, to ensure that the assessment results are robust with respect to the location of the observations, which in real problems cannot be controlled. For each set of random locations, the mean surface was calibrated assuming that the relevant terms in Equation (17) were known, but their coefficients and the type of the functional form were not. The adjusted *R* ^2^ values ranged from .952 to .971, indicating good predicting capability and stability for the regression models. The probability density functions of residuals for each set of locations and the reference PDF of the predefined fluctuations are shown to be similar in Figure 2 and the KS test for each regression model showed that the maximum value of the difference between estimate cumulative distribution functions and reference was 0.13.

The accuracy of the ACF of $g_{0}$ was tested based on the known correlation lengths (10, 12) and adjusted *R* ^2^ of the fitted surface. The number of observations were extremely limited, and thus, the number of pairs with large distances from each other was very small. This is a well‐known issue in the assessment of the ACF, which manifested itself also when using the proposed methodology. To mitigate this issue, it is customary to disregard information at distances larger than half of the investigated region. In this case, the region is 40 × 40, so common practice would lead to disregarding distances $\xi_{1}$ and $\xi_{2}$ larger than 20. Another common approach is to disregard data on pairs at a distance larger than 2 or 3 times the correlation length. In this case, the correlation lengths are easily assessed to be in the order of 10, so pairs at distances $\xi_{1}$ and $\xi_{2}$ larger than 20 or 30 would be disregarded. A sensitivity analysis was performed, considering different ranges for $\xi_{1}$ and $\xi_{2}$ and the results are collected in Table 2. The results obtained for the four different sections of location sets are very similar, so the table reports their mean values and standard deviations. The table shows that the adjusted *R* ^2^ grows when the considered distances are smaller. This reflects the fact that the observations are more abundant for pairs at short distance and the least square fitting process focuses on those values. The values associated with pairs at large distances are more scarce, the fitted surface does not describe them as accurately, and they end up compromising the value of the adjusted *R* ^2^. Instead, the assessment of the correlation distances $b_{1}$ and $b_{2}$ shows that the most accurate values are obtained when the ranges [0, 30] × [0, 30] or [0, 20] × [0, 20] are used. This is consistent with the best practices in the field, as discussed earlier. So, it is recommended that for practical applications, the upper bound of the distances to be considered for the ACF estimation is two or three times the estimated correlation lengths (as obtained with a rough preliminary assessment).

**TABLE 2:** Goodness of fit in ACF with no noise, expressed as mean ± standard deviation.

Limits for $\xi_{1}$ and $\xi_{2}$	*b*_1_ (reference value: 10)	*b*_2_ (reference value: 12)	Adjusted *R* ^2^
[0, 40] × [0, 40]	10.22 ± 1.64	13.71 ± 0.63	0.059 ± 0.00
[0, 30] × [0, 30]	10.11 ± 0.65	11.73 ± 1.15	0.082 ± 3.77 × 10^−4^
[0, 20] × [0, 20]	10.03 ± 0.86	10.82 ± 0.69	0.14 ± 3.16 × 10^−4^
[0, 10] × [0, 10]	9.59 ± 0.77	11.69 ± 2.44	0.28 ± 0.00
[0, 5] × [0, 5]	9.58 ± 1.41	11.57 ± 2.09	0.44 ± 0.015

After the first calibration was completed, the study was repeated with increasing levels of noise z.

**FIGURE 3:**
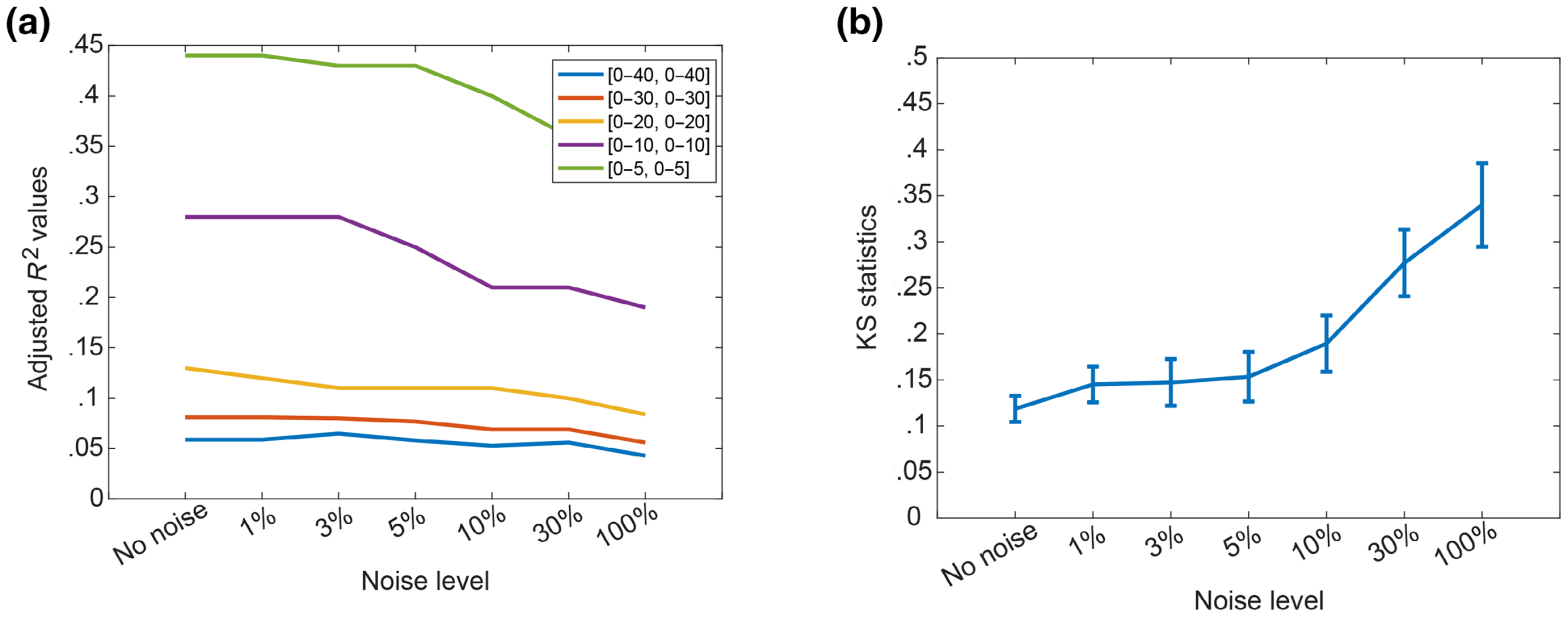
The mean values of adjusted *R* ^2^ for ACF out of four sets of locations with various noise levels (a), KS statistics with various noise levels (b).

**FIGURE 4:**
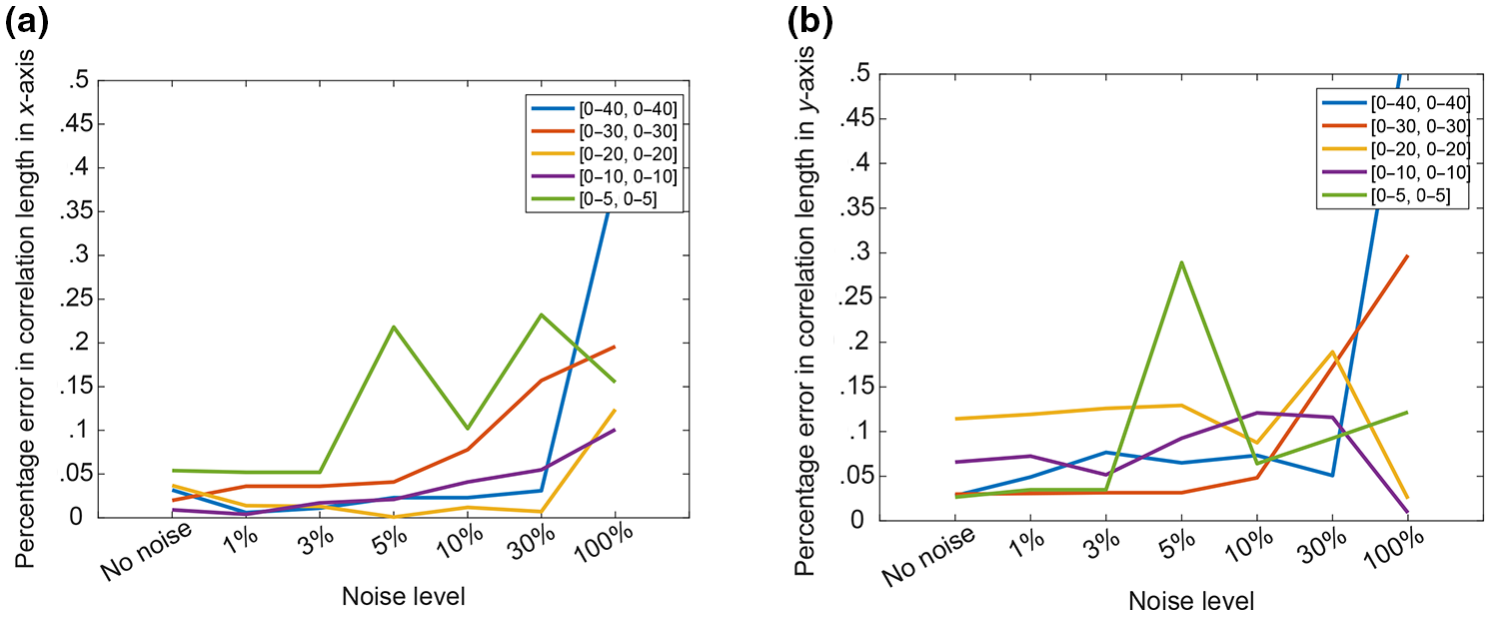
Percentage errors in the estimates of the correlation lengths along *x*‐axis (a), and along *y*‐axis (b).

Figure 3b shows the results in terms of the ability to capture the marginal distribution of the investigated field, as measured by the KS index. The results shown in the figure are averaged for the four different sets of random locations. The results show that the accuracy is not compromised by measurement noise up to 3%, and generally acceptable for noise up to 10%. For even higher levels of noise, the KS index exceeded 0.25, and it is deemed unacceptable. The accuracy in the estimation of the ACF was determined based on the adjusted *R* ^2^ of the fitted surface and the percentage error in the correlation lengths, computed as:

(18) $${\text{Error}}_{i}=\frac{{\hat{b}}_{i}-b_{i}}{b_{i}}$$

where $b_{i}$ is the reference value of the correlation length in the i‐th dimension (known in this example to be 10 and 12) and ${\hat{b}}_{i}$ is the estimated value for correlation length in i‐th dimension when noise is introduced. Figure 3a shows that the adjusted *R* ^2^ is not very sensitive to the noise level, especially for the cases with the recommended focus for $\xi_{1}$ and $\xi_{2}$ in [0, 20] or [0, 30]. Figure 4 shows that the error in the correlation lengths increases sharply when a noise level is more than 30% (Figure 4). Overall, these results show that the assessment methodology is quite robust to noisy data, even for noise levels up to 30% of the fluctuations.

### 3.2 The bare carrying capacity

The variables ${\mathrm{POP}}_{n}$, ${\mathrm{LAT}}_{n}$, and ${\mathrm{LON}}_{n}$ were found to be contributing in a negligible way to the accuracy of each type of regression model. To confirm that they provide only redundant information, the adjusted *R* ^2^ values for the models was computed twice, once with all terms and once excluding the terms including those three variables. The results are collected in Table 3. By excluding ${\mathrm{LAT}}_{n}$, ${\mathrm{LON}}_{n}$, ${\mathrm{POP}}_{n}$, the loss in accuracy is minimal, thus for the search of the best subset, those three variables were removed from the dataset. Because there is a significant improvement (see Table 3) in the model accuracy by including cross terms and only a slight improvement further including the other second degree terms, the GLC model type was chosen for estimating μ, as the best trade‐off between accuracy and complexity of the model. For each model type, it was found that in the cross‐validation, the mean adjusted *R* ^2^ increased considerably when the first four variables were included, and then became almost constant.

**TABLE 3:** Adjusted *R* ^2^ values.

Adjusted *R* ^2^	MLR	GLC	GPC
All variables	0.6134	0.7718	0.7834
Excluding ${\mathrm{POP}}_{n}$, ${\mathrm{LAT}}_{n}$, and ${\mathrm{LON}}_{n}$	0.6133	0.7658	0.7775

**FIGURE 5:**
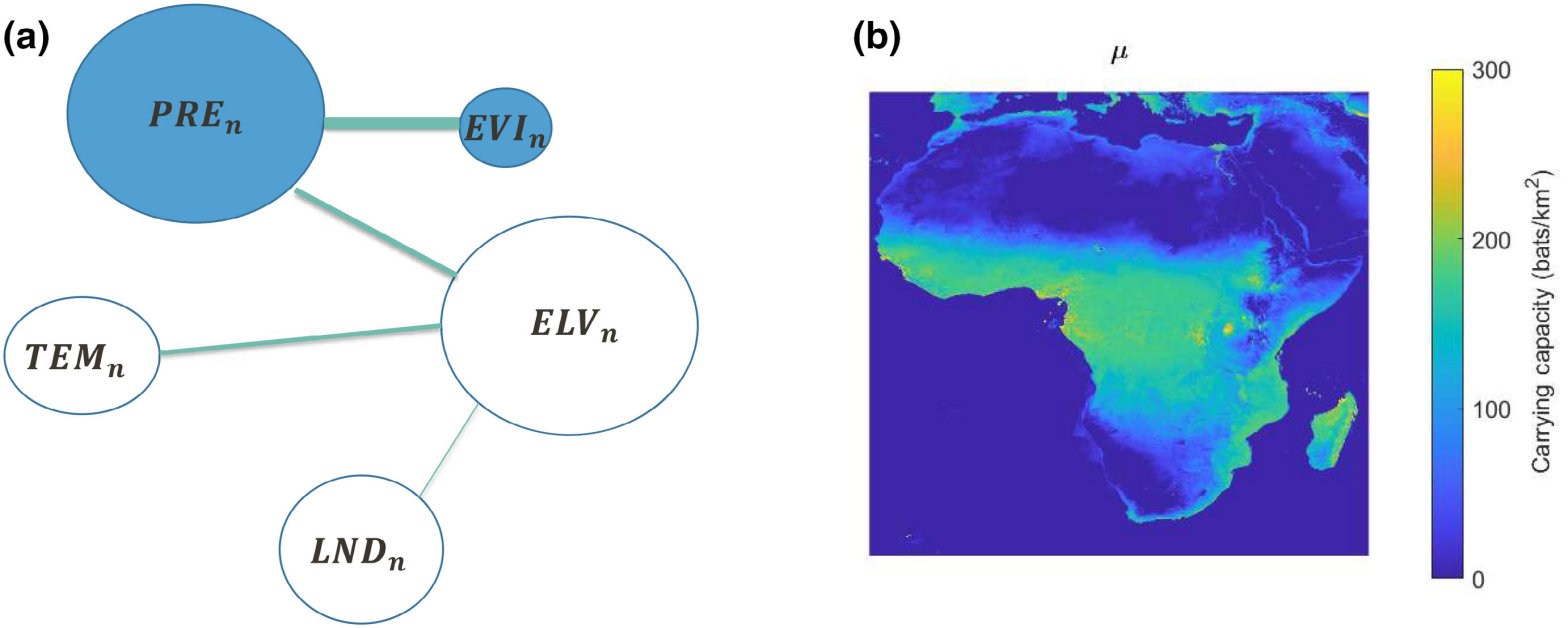
Visual representation of model mean surface of the bare carrying capacity μ (a), Mean bare carrying capacity using the expression in Equation (19) (b).

The shape of the distribution of the adjusted *R* ^2^ obtained for different partitioning of the dataset between training and testing was not substantially affected by the number of variables. The analysis using *regsubsets* identified a model with only six terms containing a combination of all five environmental variables (i.e., ${\mathrm{EVI}}_{n}$, ${\mathrm{ELV}}_{n}$, ${\mathrm{PRE}}_{n}$, ${\mathrm{TEM}}_{n}$, and ${\mathrm{LND}}_{n}$) and yielding good accuracy. For this reason, such model was selected for estimating μ. Equation (19) shows the final model and Figure 5a provides its pictorial representation (see folder S3 in Mursel et al., 2023). The blue circles represent the included variables, and the edges represent the included cross terms; the size (i.e., the area) of the circles and the thickness of the edges are proportional to the weight of each term. The estimated mean surface $\hat{\mu }$ computed across Africa is presented in Figure 5b.
(19)

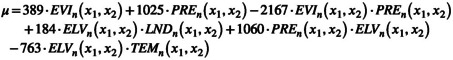




**FIGURE 6:**
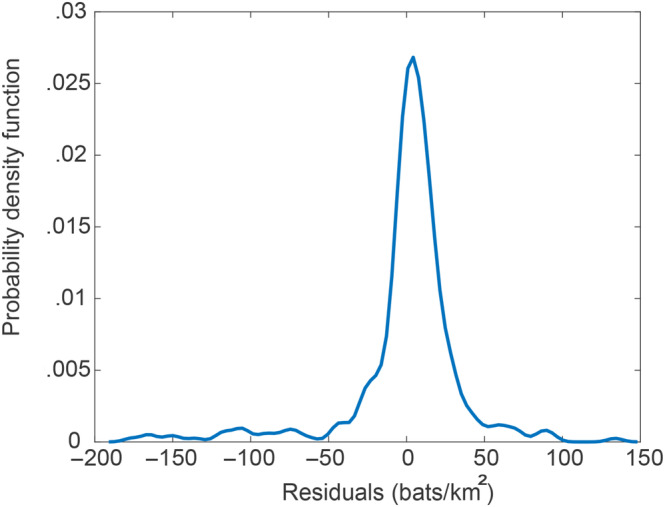
Probability density function of the residuals $f_{\mathrm{obs}}\left(x_{1},x_{2}\right)$.

The differences between values estimated through Equation (19) and the actual values of bare carrying capacity observations are the residuals $f_{\mathrm{obs}}$. These values constitute a sample of the random field of residuals $f_{0}\left(x_{1},x_{2}\right)$ across the continent of Africa. The statistical characteristics of the sample can be computed. For instance, Figure 6 shows the probability density function, demonstrating that there are fluctuations around the mean surface at all scales, but most residuals are small, relative to the values of $\hat{\mu }$.

**FIGURE 7:**
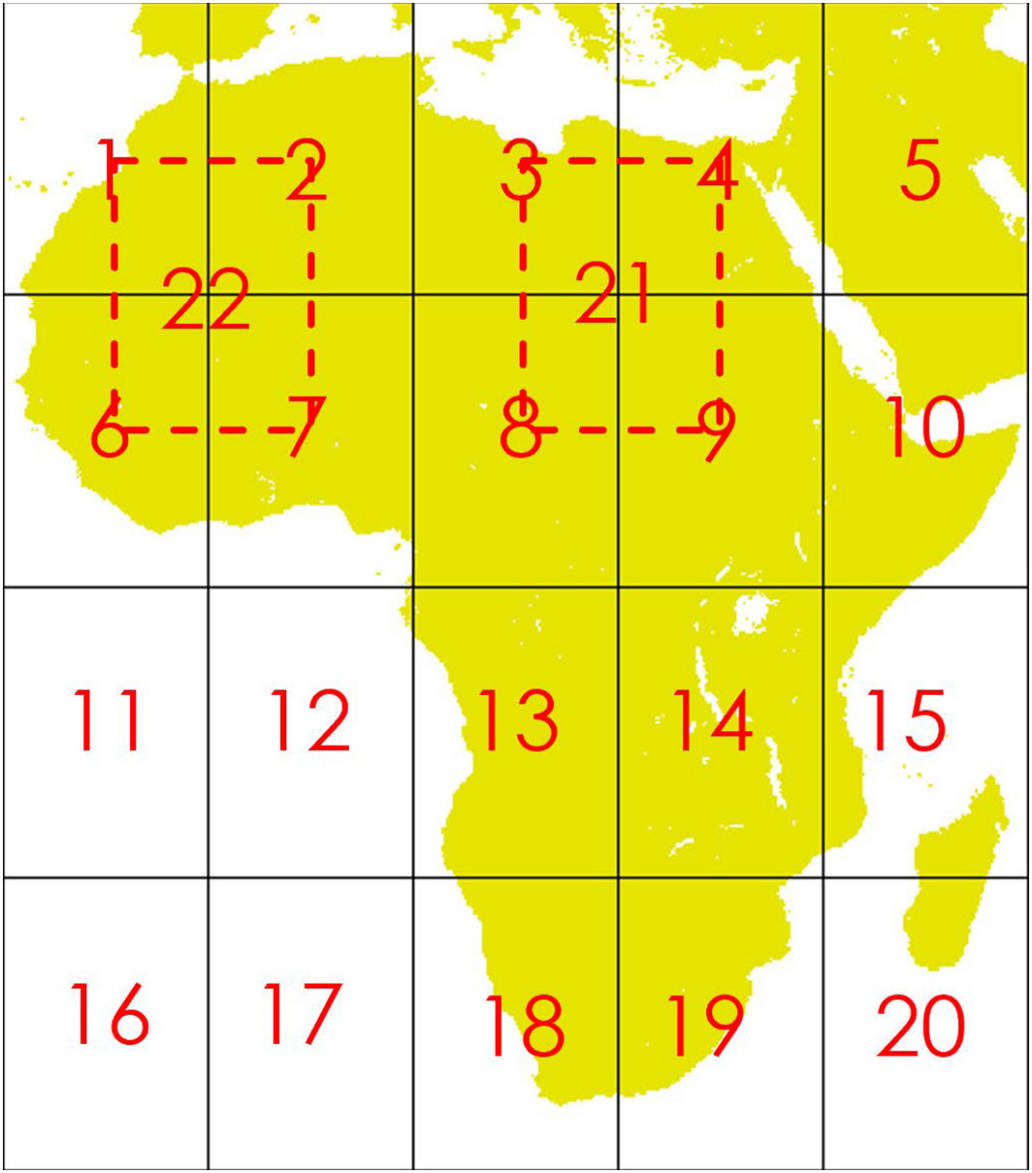
Subregions to test homogeneity across Africa.

**FIGURE 8:**
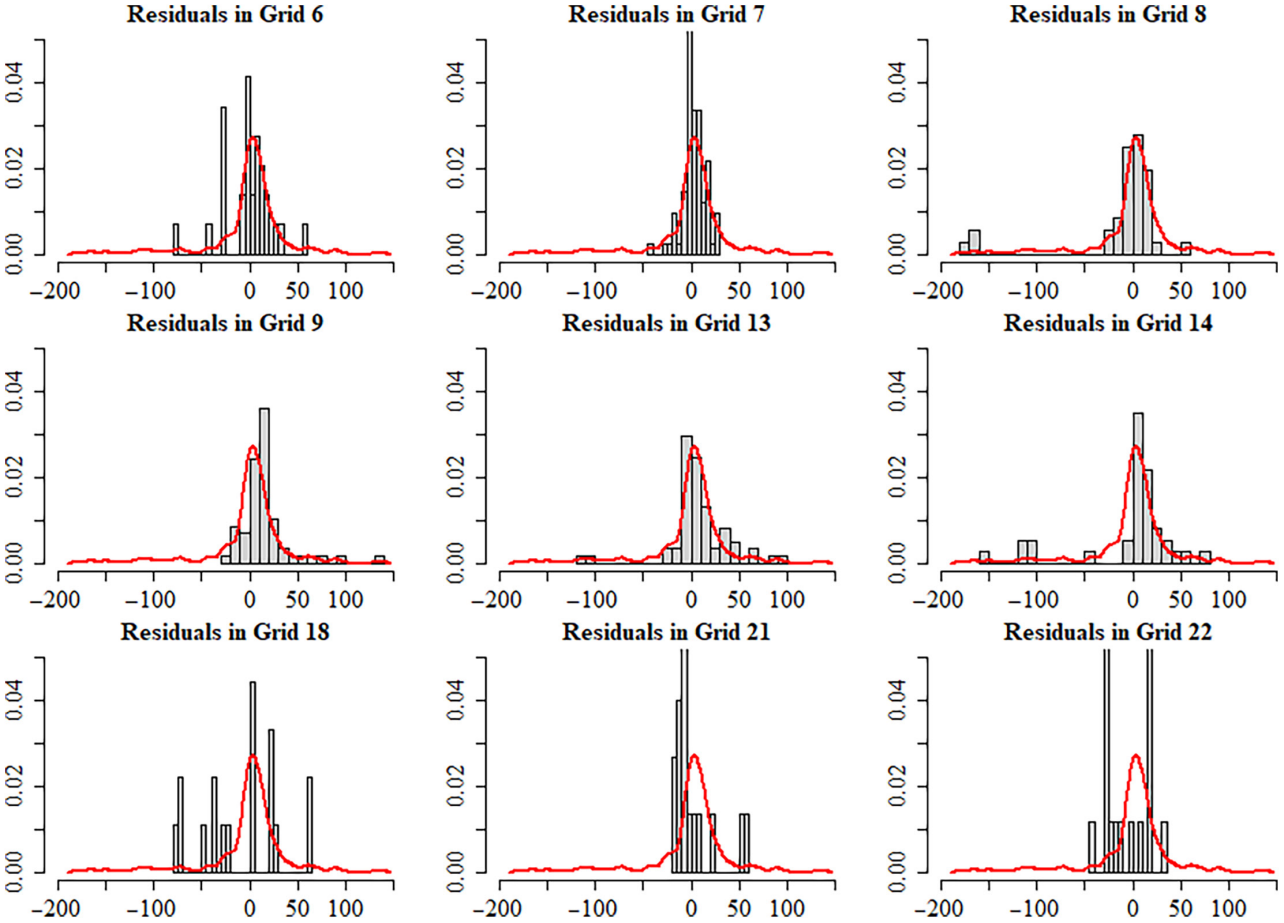
Distributions of subsamples at different locations. All values in the horizontal axis are in bats/km^2^. Histograms show the samples located in corresponding subregions and red curve represents the probability distribution of the whole sample resulting from the kernel smoothing.

The mean of the residuals is 0 bats/km^2^ by construction, and its standard deviation is 36.9 bats/km^2^. The *p*‐value from the Shapiro–Wilk normality test is .0051, which is far below the common threshold for acceptability of .05, implying that the data significantly deviate from a Gaussian distribution. The test of homogeneity consisted in checking if the statistical properties depend upon the spatial coordinates. Therefore, the continent of Africa was split into 20 smaller subregions (14° × 20°) and probability distributions of the values of the sample in the various subregions were compared to each other and to the probability distribution of values in the whole sample. Two overlapping subregions were also included to capture the data points in some overlapping sections (subregions 21 and 22 in Figure 7). The size of the subregions was selected in such a way to balance the needs of a sufficiently high resolution over the continent, and of having at least 10 values in each subregion to compute the statistics. The homogeneity check confirmed that the statistical characteristics of the sample are independent on the spatial location. In fact, Figure 8 shows the probability density function of the residuals at all cells, except for those with less then 10 data points, which were not included in this analysis.

While the shape of the probability distribution resulting from kernel smoothing is very similar in all cells, the relative frequency histograms for cells 21 and 22 indicate a slightly different distribution of the residuals. This indicates that the assumption of homogeneity is less accurate for cells that are almost entirely centered on the Sahara desert, where all observations are 0 bats/km^2^. To address this, the analysis could be carried out focusing exclusively on the portion of Africa South of the Sahara Desert. However, in this study, we decided to study the entire continent, rely on the fact that the kernel smoothing results are consistent across all cells, and accept the fact that the results may be slightly less accurate over Sahara. Due to the non‐Gaussianity of the field, the values of $f_{\mathrm{obs}}\left(x_{1},x_{2}\right)$ were mapped into a corresponding (“underlying”) Gaussian distribution using Equation (4).

The surface $R_{\mathrm{obs}}$ computed with Equation (5) diminishes with increasing distances (i.e., $\xi_{1},\xi_{2}$), as expected. The values of the $R_{\mathrm{obs}}$ for distances larger than 20°, which corresponds to 2220 km on average, in both directions where disregarded for ecological and numerical reasons. From an ecological point of view, the correlation of available resources in the environment at a distance of 2000 km seems impossible to justify. From a numerical point of view, the values of $R_{\mathrm{obs}}$ are very sparse at these distances, and they oscillate around a zero mean, reinforcing the notion that they are just numerical artifacts.

**FIGURE 9:**
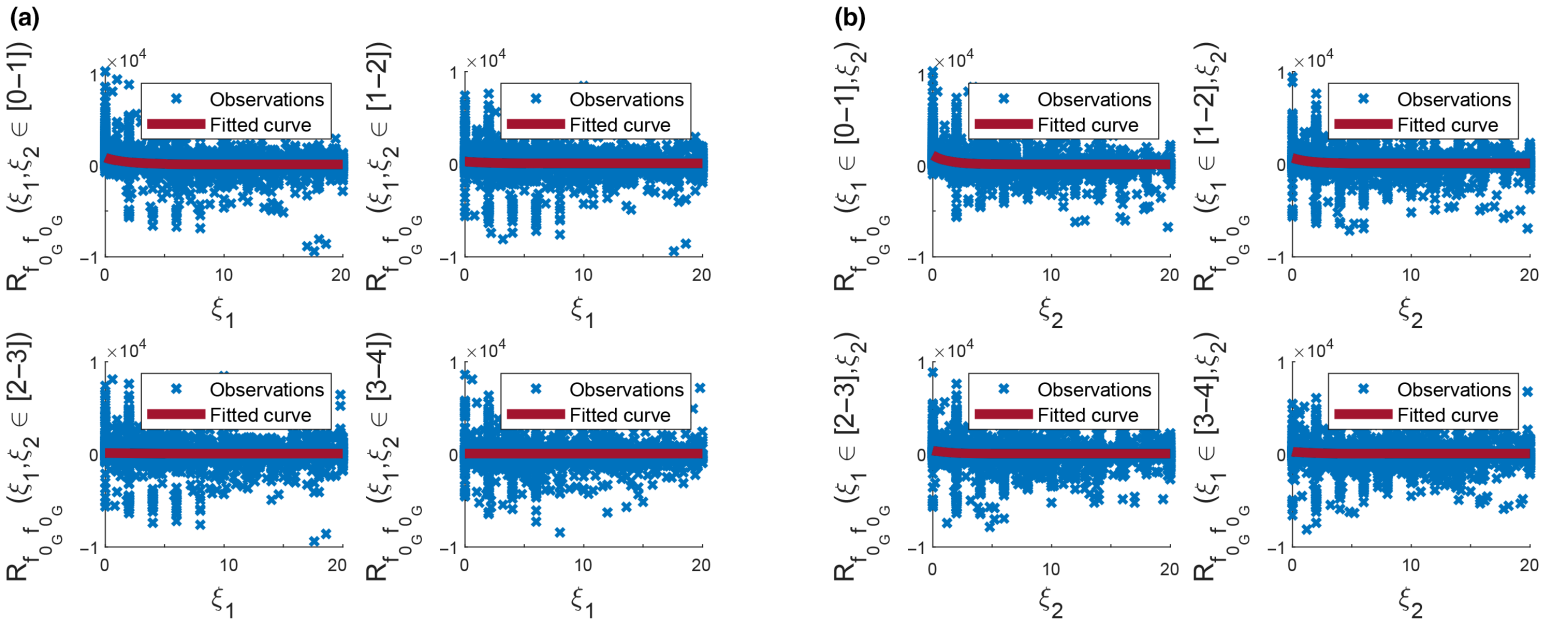
Comparison of EACF and analytical ACF along the longitude (a), along the latitude (b).

**FIGURE 10:**
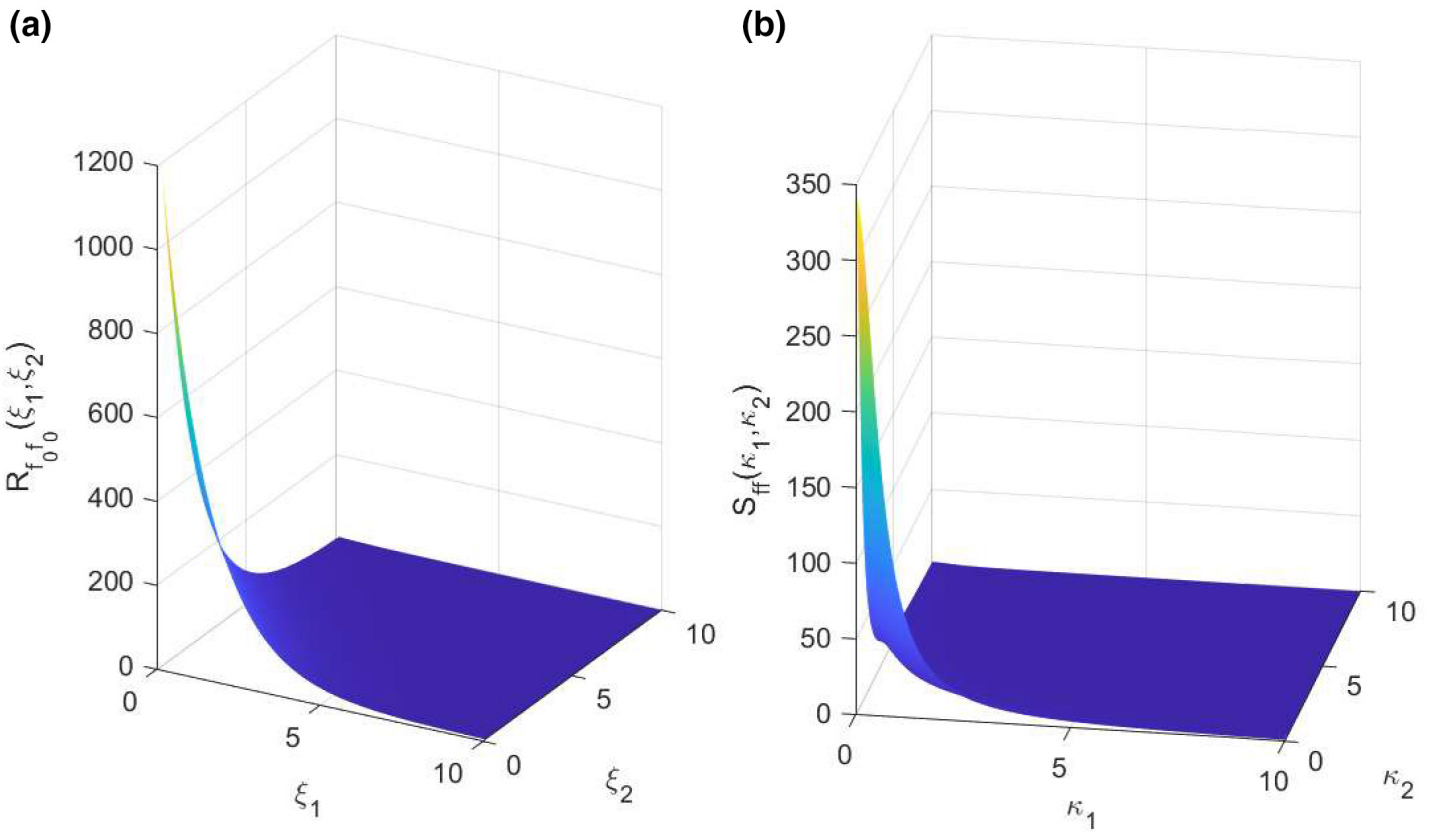
Analytical forms of ACF (a), and spectrum (b).

**FIGURE 11:**
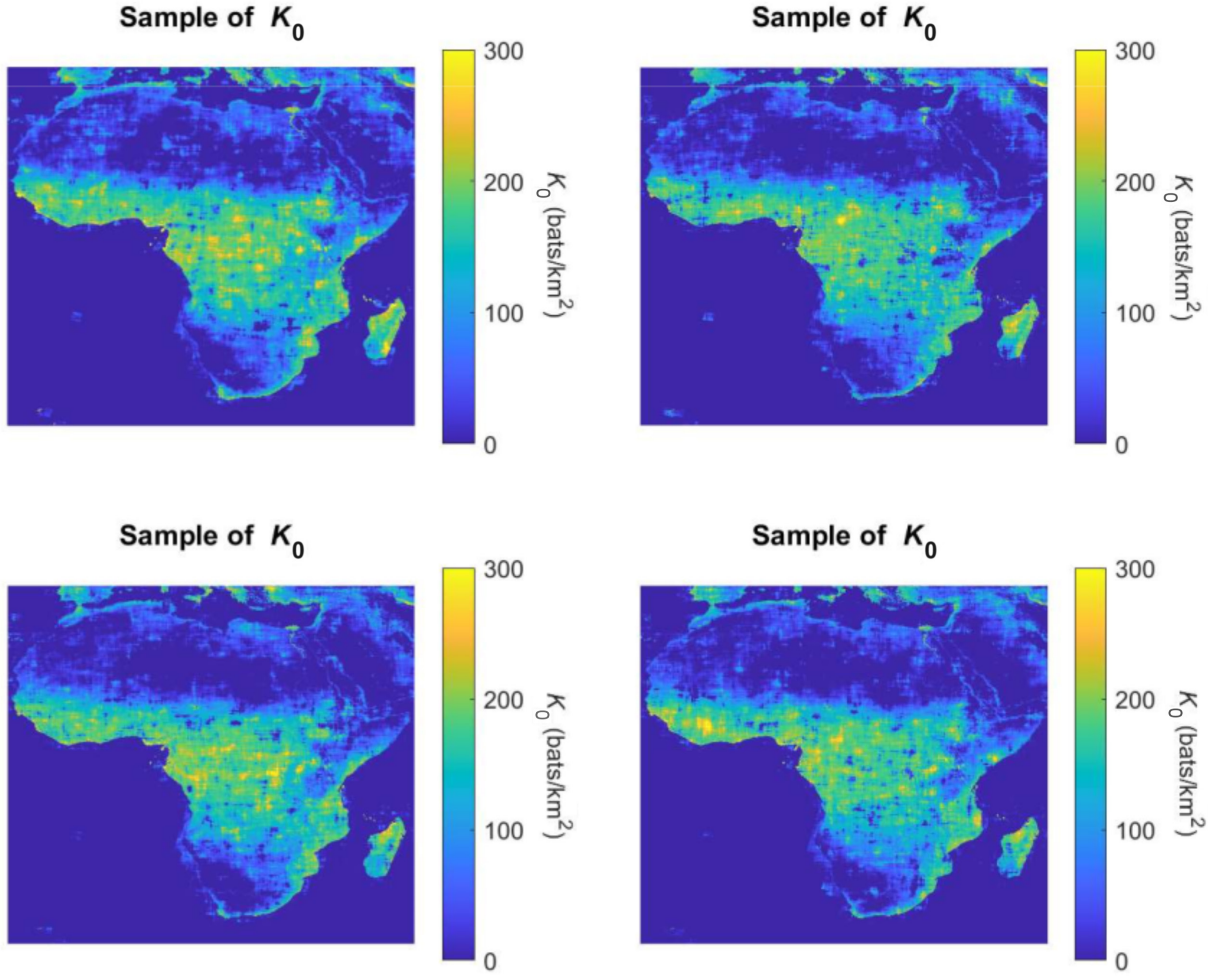
Four generated samples of $K_{0}$ across Africa.

Therefore, the candidate functional forms were fitted only considering the lag interval [0°, 20°] ×[0°, 20°]. A set of decaying functional forms often used to describe the ACF were fitted and, as a metric of accuracy, their adjusted *R* ^2^ were compared. In addition, at some arbitrarily chosen discrete lines (e.g., $\xi_{1}=0.5$, $\xi_{2}=2.5$), the trends of the $R_{\mathrm{obs}}$ and the fitted surfaces were compared visually, to assess qualitatively the potential functional forms as well. At the end, an exponentially decaying function provided the largest adjusted *R* ^2^ among all candidate functional forms, and it was found to be convincing by visual inspection. Hence, the ACF of the residuals of the bare carrying capacity was assumed to follow the functional form in Equation (20):

(20) $${\hat{R}}_{f_{0}f_{0}}\left(\xi_{1},\xi_{2}\right)=s^{2}\exp\left(-\left|\frac{\xi_{1}}{b_{1}}\right|-\left|\frac{\xi_{2}}{b_{2}}\right|\right)$$

(21) $${\hat{S}}_{f_{0}f_{0}}\left(\kappa_{1},\kappa_{2}\right)=\frac{s^{2}\cdot b_{1}\cdot b_{2}}{\pi^{2}\cdot \left(1+b_{1}^{2}\kappa_{1}^{2}\right)\cdot \left(1+b_{2}^{2}\kappa_{2}^{2}\right)}$$

where s is the standard deviation of the field, $b_{1}$ and $b_{2}$ are correlation lengths along longitude and latitude, respectively. The correlation lengths, $b_{1}$ and $b_{2}$, for the best fitted curve were found to be 1.779° and 1.395°, respectively. The power spectral density function in Equation (21) completes the well‐known pair, which could also be found using the Wiener–Khinchin theorem in Equation (6). The functions in Equations (20) and (21) were used to characterize the behavior of the underlying Gaussian random field and generate Gaussian samples across the space. For representation purposes, $R_{\mathrm{obs}}$ and the fitted analytical form ${\hat{R}}_{f_{0}f_{0}}$ at 4 lags in longitude and latitude are presented in Figure 9. It is concluded that this functional form is a good representation of the ACF, and the resulting autocorrelation and spectrum are shown in Figure 10. These functions were used in the SRM algorithm and samples representing the residuals were generated. Gaussian samples were translated into the non‐Gaussian distribution (Figure 6) calibrated empirically (see folder S1 in Mursel et al., 2023). Some samples superimposed to $\hat{\mu }$ are presented in Figure 11.

## 4 DISCUSSION AND CONCLUSIONS

Our aim was to develop a new technique to calibrate the model of a random field when data are available only on an irregular grid. With the proposed procedure, it is possible to have an accurate estimation of the ACF even when large gaps in the data are present. The numerical example shows that even with as few as 100 observations of a single sample of the field, with relatively large gaps, the proposed method still gives a satisfactory approximation of the first and second order statistical characteristics of the field. The same example shows that the method can tolerate levels of noise as large as 30% and still provide results that can be sufficiently accurate to drive decision‐making. This directly means that the methodology is robust against considerable amount of imperfections in the model, approximations, and measurement errors.

### 4.1 Model of bare carrying capacity of bats

The possible relationship between Ebola (and likely other zoonotic diseases) and bats has increased the attention to models of dynamics of bat species, as well as their relationship with the surrounding environment. To address this problem, studies based on dynamics of virus transmission among bats (e.g., SIR models, agent‐based models) needs to be combined with models of the animal behavior and migration patterns (Fiorillo et al., 2018). These models are driven by the values of the carrying capacity $K_{0}$, which cannot be directly measured, hence the need for the presented probabilistic model that enables the generation of samples that capture the global trend, as well as local random fluctuations. Herein, 10^5^ different realizations of bare carrying capacity at the resolution of 10 km × 10 km were generated, after the calibration of the random field. The randomness in the model is due to several reasons. First, the regression model for the mean value μ for the bare carrying capacities is only an approximation of the general behavior and it is not perfectly accurate. This is partly due to the input data, which included only a few of the many factors that affect the natural bare carrying capacity of a location. In addition, the values used for the environmental data were averaged over 10 years. Moreover, even the basic hypothesis that the bare carrying capacity is a function of environmental parameters is reasonable, yet it is not strictly correct. In fact, in addition to environmental parameters, there are other factors that could be relevant, such as predator–prey relationships, as well as the presence of humans and of industrial activities. These other factors are not taken into account in this study, through Equation (19).

Regarding the regression model for the estimation of mean bare carrying capacity, it should be noted that it could predict negative values at some locations, as there is no predefined positivity constraint. However, this quantity in nature cannot take a negative value. Moreover, even where the mean predicted value of the bare carrying capacity μ is positive, the random fluctuation $f_{0_{G}}$ can take a large negative value and their superposition can lead to negative values of $K_{0}$ at some locations and for some generated samples. To address this issue, we simply set the value of $K_{0}$ = 0 bats/km^2^ at all the locations with negative bare carrying capacity in the post‐processing phase.

**FIGURE 12:**
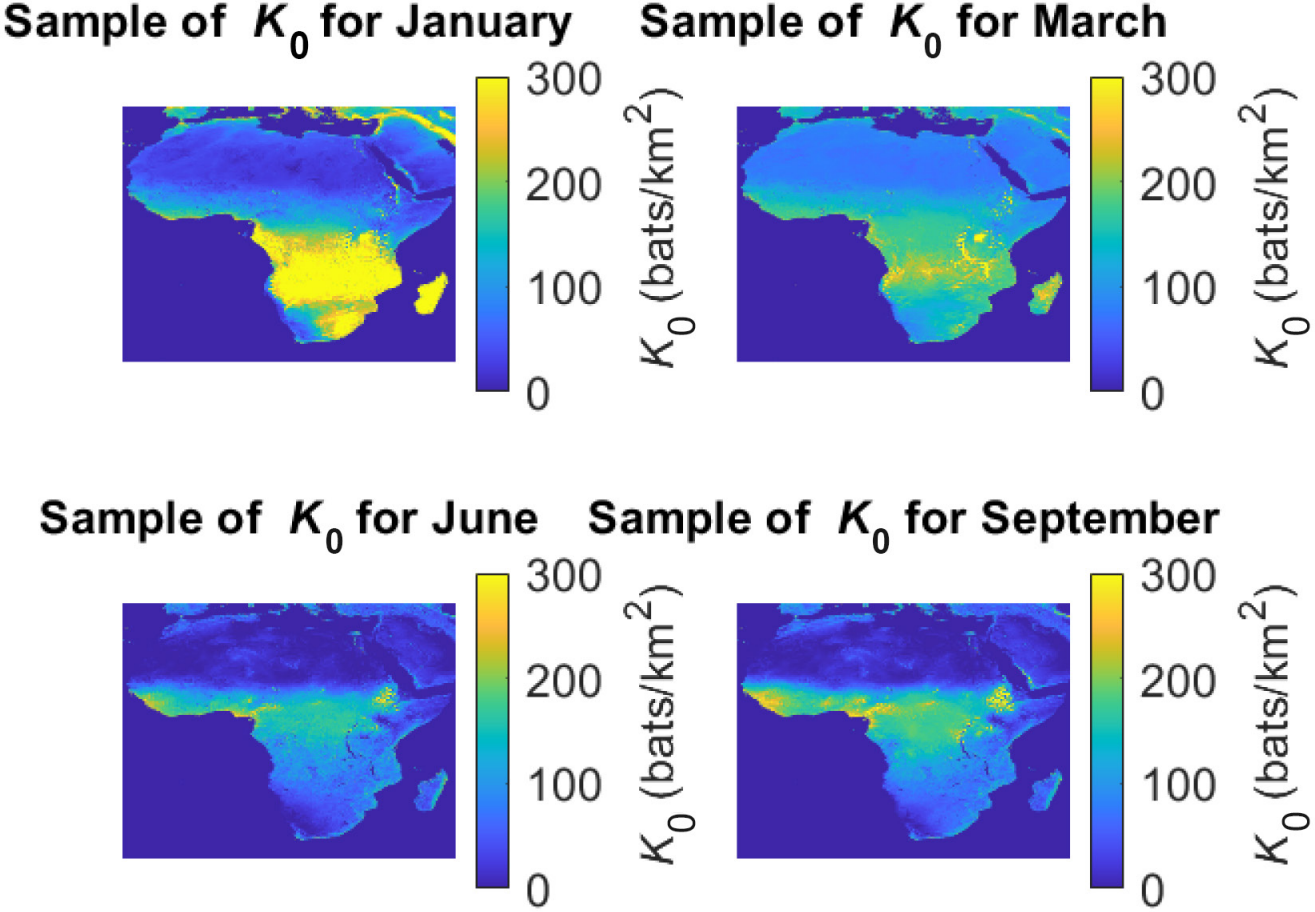
Mean surface of $K_{0}$ across Africa for different seasons.

In the modeling phase, we combined environmental data at all times of the year, even though seasonality has direct impacts on the environmental variables. This was done because the data on bats presence/absence are not accompanied by timestamps, so they could not be associated with a specific season of the year, they should be interpreted as annual values. Thus, for the regression analysis, we decided to be consistent and use annual averages also for the environmental variables. However, for the sake of investigating seasonal variability, we computed also month‐specific regression models of μ for the bare carrying capacities. To do this, we kept the same data on bat presence and absence, but for the input variables (e.g., precipitation), we used for each month the average of the environmental parameters of that single month over 10 years (from 2000 to 2010). On the resulting monthly average surfaces, we superimposed samples of the same random fluctuations calibrated in Section 2.3 to obtain samples of the monthly values. Figure 12 shows samples for selected months (see folder S2 in Mursel et al., 2023).

The correlation lengths for the sample was found to be 1.779° and 1.395°, along longitude and latitude, respectively (Figure 11). These values correspond to approximately 198 km along the longitude and 155 km along the latitude, with the actual values slightly changing for different parts of the globe. This outcome is not surprising, as all the environmental parameters that were not directly included in Equation (19) change more rapidly along the longitude than along the latitude. Just like the climate, also the vegetation is more prone to change drastically along the latitude, so the correlation in the fluctuations of the bare carrying capacity should be short. Instead, along the longitude, the environmental variables change more gradually and the correlation length is expected to be larger.

It should be noted that the values of the correlation lengths $b_{1}$ and $b_{2}$ were found for the underlying Gaussian field, and not directly for the bare carrying capacity. It is well known that the nonlinear transformation in Equation (12) warps the ACF and the spectrum of the resulting field, and this effect can be computed in closed form (Grigoriu, 1995). This effect was counteracted by pre‐mapping the observation to the underlying Gaussian counterpart, with Equation (4), so that the resulting non‐Gaussian samples match the probabilistic characteristics of the observations. However, the fact remains that the values of 198 and 155 km for the correlation lengths along longitude and latitude would be slightly different after mapping.

### 4.2 Observations on the ecology of the bare carrying capacity

As Figure 11 shows, $K_{0}$ in central Africa has some dense spots. The yellow regions, which correspond to higher bare carrying capacity, can be found especially in the West coast of Africa. These spots in West Africa (e.g., Guinea, Sierra Leone) lie in the regions where the Ebola virus disease caused the most severe and widespread consequences during the 2014–2016 outbreak (Chan, 2014). This could be an indicator that our model can indeed identify regions with high potential density of infected animal carriers. Also, our results suggest that regions with high bare carrying capacity are not limited to West Africa but are present also along the West coasts of Central Africa, including Gabon, Democratic Republic of Congo and Cameroon. This could be a reason of concern and provide an alert for the government of Cameroon, because Cameroon has not yet been affected by Ebola virus (Akem & Pemunta, 2020). However, the carrying capacity is not sufficient to determine areas at risk, the dynamics of bat migrations and the propensity of the population in engaging in behaviors that expose them to contacts with wildlife need to be studied to obtain a holistic assessment of risk.

Our results also point that the Itombwe Massif in the Democratic Republic of Congo is another region that can attract and support bats. This result is consistent with the fact that the Itombwe Massif has been recognized as one of the most biologically diverse regions in Africa (Omari et al., 1999) and it is reasonable to infer that it provides resources that support the nesting, foraging, and reproduction of bats. These results suggest that our estimates can indeed identify the regions that are known/anticipated to be most populated by bats.

**Nomenclature**
x
: Spatial parameter
θ
: Outcome
Ω
: Spatial domain
Θ
: Sample space of outcomes
$f_{0}$
: Zero mean random field
$b_{i}$
: Correlation length
$R_{f_{0}f_{0}}$
: Auto‐correlation function
$\xi_{1},\xi_{2}$
: Spatial lags (differences between spatial parameters, x) in the two investigated dimensions
μ
: Mean surface
$g_{0}$
: Random field
$f_{0_{\mathrm{NG}}}$
: Non‐Gaussian random field
$F_{G}$
: Inverse standard Gaussian cumulative distribution function
$\hat{F_{\mathrm{NG}}}$
: Inverse empirical cumulative distribution function
$R_{e\;f_{0}f_{0}}$
: Empirical estimate of autocorrelation function (EACF) of the field
${\hat{R}}_{f_{0}f_{0}}$
: Fitted surface of estimate of autocorrelation function of the field with its analytical expression
${\hat{S}}_{f_{0}f_{0}}$
: Fitted surface of estimate of spectral density function of the field with its analytical expression
$\kappa_{1},\kappa_{2}$
: Wave number in the two investigated dimensions
$X_{1},X_{2},X_{3},X_{4}$
: Fully known surfaces
$f\left(x_{1},x_{2}\right)$
: Single sample of a two‐dimensional random field
$g_{0}\left(x_{1},x_{2}\right)$
: Benchmark random field
$g\left(x_{1},x_{2}\right)$
: Single sample of benchmark random field $g_{0}$
${\tilde{x}}_{1},{\tilde{x}}_{2}$
: Coordinates of a small subset of randomly selected grid points
z
: Gaussian noise
$g_{z}$
: Superposition of sample of a random field g and noise z
$\hat{f}$
: Residuals
$\hat{\mu }$
: Estimate of mean surface μ
$K_{0}$
: The bare carrying capacity
$K_{0\;\mathrm{obs}}$
: The bare carrying capacity where observations are available
EVI
: Enhanced vegetation index
PRE
: Precipitation
TMP
: Daily air temperature
LND
: Land cover index
ELV
: Ground elevation
POP
: Human population
LAT
: Latitude
LON
: Longitude
$\phi_{n_{1}n_{2}}^{\left(1\right)},\phi_{n_{1}n_{2}}^{\left(2\right)}$
: Independent random phase angles uniformly distributed over the interval $\left[0,2\pi \right]$
$\kappa_{1u},\kappa_{2u}$
: Upper cutoff wave numbers
$\bar{b_{1}},\bar{b_{2}}$
: Reference values of correlation lengths in investigated dimensions

## AUTHOR CONTRIBUTIONS

**Sena Mursel:** Conceptualization (equal); data curation (equal); formal analysis (equal); methodology (equal); software (equal); writing – original draft (equal). **Daniel Conus:** Conceptualization (equal); methodology (equal); supervision (equal); writing – review and editing (equal). **Wei‐Min Huang:** Conceptualization (equal); methodology (equal); supervision (equal); writing – review and editing (equal). **Javier Buceta:** Conceptualization (equal); funding acquisition (equal); methodology (equal); supervision (equal); writing – original draft (equal). **Paolo Bocchini:** Conceptualization (equal); funding acquisition (equal); methodology (equal); project administration (equal); supervision (equal); writing – review and editing (equal).

## FUNDING INFORMATION

The financial support of the US National Institute of Health through grant 1R15GM123422‐01A1 (“Risk Assessment of Ebola Outbreaks through Probabilistic Modeling of Chiroptera Zoonotic Dynamics and Socioeconomic Factors”) and of Lehigh University through the “Research Futures: Major Program Development” grant (“Catastrophe Modeling for Natural Disasters and Health Related Threats”) is gratefully acknowledged.

## CONFLICT OF INTEREST STATEMENT

The authors declare no conflicts of interest.

## Data Availability

Matlab scripts and data files to perform the analyses are supplied as supplementary materials at the Figshare Data repository (Mursel et al., 2023).
